# Implementing recommendations for inpatient healthcare provider encouragement of cardiac rehabilitation participation: development and evaluation of an online course

**DOI:** 10.1186/s12913-020-05619-2

**Published:** 2020-08-20

**Authors:** Carolina Santiago de Araújo Pio, Anna Gagliardi, Neville Suskin, Farah Ahmad, Sherry L. Grace

**Affiliations:** 1 York University, Toronto, ON Canada; 2 University Health Network, University of Toronto, Toronto, ON Canada; 3 Western University, London, ON Canada; 4 St. Joseph’s Health Care London, London, ON Canada

**Keywords:** Cardiac rehabilitation, Health services, Nursing, Patient participation, Professional education

## Abstract

**Background:**

A policy statement recommending that healthcare providers (HCPs) encourage cardiac patients to enroll in cardiac rehabilitation (CR) was recently endorsed by 23 medical societies. This study describes the development and evaluation of a guideline implementation tool.

**Methods:**

A stepwise multiple-method study was conducted. Inpatient cardiac HCPs were recruited between September 2018–May 2019 from two academic hospitals in Toronto, Canada. First, HCPs were observed during discharge discussions with patients to determine needs. Results informed selection and development of the tool by the multidisciplinary planning committee, namely an online course. It was pilot-tested with target users through a think-aloud protocol with subsequent semi-structured interviews, until saturation was achieved. Results informed refinement before launching the course. Finally, to evaluate impact, HCPs were surveyed to test whether knowledge, attitudes, self-efficacy and practice changed from before watching the course, through to post-course and 1 month later.

**Results:**

Seven nurses (71.4% female) were observed. Five (62.5%) initiated dialogue about CR, which lasted on average 12 s. Patients asked questions, which HCPs could not answer. The planning committee decided to develop an online course to reach inpatient cardiac HCPs, to educate them on how to encourage patients to participate in CR at the bedside. The course was pilot-tested with 5 HCPs (60.0% nurse-practitioners). Revisions included providing evidence of CR benefits and clarification regarding pre-CR stress test screening. HCPs did not remember the key points to convey, so a downloadable handout was embedded for the point-of-care. The course was launched, with the surveys. Twenty-four HCPs (83.3% nurses) completed the pre-course survey, 21 (87.5%) post, and 9 (37.5%) 1 month later. CR knowledge increased from pre (mean = 2.71 ± 0.95/5) to post-course (mean = 4.10 ± 0.62; *p* ≤ .001), as did self-efficacy in answering patient CR questions (mean = 2.29 ± 0.95/5 pre and 3.67 ± 0.58 post; *p* ≤ 0.001). CR attitudes were significantly more positive post-course (mean = 4.13 ± 0.95/5 pre and 4.62 ± 0.59 post; *p* ≤ 0.05). With regard to practice, 8 (33.3%) HCPs reported providing patients CR handouts pre-course at least sometimes or more, and 6 (66.7%) 1 month later.

**Conclusions:**

Preliminary results support broader dissemination, and hence a genericized version has been created (http://learnonthego.ca/Courses/promoting_patient_participation_in_CR_2020/promoting_patient_participation_in_CR_2020EN/story_html5.html). Continuing education credits have been secured.

## Background

Cardiovascular diseases (CVDs) are among the leading burdens of disease and disability worldwide [1, 2]. In 2015, there were 422.7 million CVD cases globally [3], and these patients are at high risk of recurrent cardiac events and death [2]. Thus, secondary prevention is needed [4].

Cardiac Rehabilitation (CR) is a proven, cost-effective, outpatient model of care comprised of structured exercise training, patient education and counselling, as well as risk factor management [5, 6]. The benefits of CR include 20% reductions in morbidity and CV mortality [7]. Despite the benefits, CR utilization is low [8–12]. One of the main reasons is lack of referral and encouragement by healthcare providers (HCPs) [13].

The recent update of the Cochrane Collaboration review on interventions to promote CR utilization undertaken by our group [14] identified effective strategies. Findings included that CR enrolment is significantly greater when an intervention is delivered by a healthcare provider (HCP), face-to-face. This could be undertaken most feasibly and affordably through communication at the bedside with CV inpatients prior to hospital discharge. A position statement to forward recommendations based on the findings was subsequently developed, and endorsed by 23 medical societies [15, 16].

However, the development of a guideline or position statement is insufficient to change clinical practice and hence achieve greater patient utilization; therefore implementation tools are needed [17]. Indeed, a 2016 Cochrane review showed that implementation tools developed and disseminated with guidelines positively influence clinician behavior and patient outcomes [18]. Accordingly, implementation tools are recommended in standards for guideline development [19–21].

We undertook an environmental scan and consulted experts globally through the International Council of Cardiovascular Prevention and Rehabilitation (ICCPR), and could not identify tools to support implementation of the recommendations (what is available is shown here: http://sgrace.info.yorku.ca/tools-to-promote-cardiac-rehabilitation-utilization/). Thus, a needs assessment was undertaken to discern the best type of tool(s) to promote patient-provider bedside discussions regarding CR; results were used to develop and then test a guideline implementation tool. The objectives of this paper were to describe the needs assessment, implementation tool development process, and evaluation of its’ efficacy, with regard to learner knowledge, attitudes, self-efficacy, and practice. This tool could improve awareness and discussion about, as well as utilization of, CR, leading to greater secondary prevention of CVD.

## Methods

### Team composition and stakeholder engagement

The team / planning committee was comprised of the co-chairs of the policy statement on interventions to increase CR utilization (CSAP, SLG) [15, 16], as well as the methodologist with expertise in guideline implementation (AG). Clinicians who would be implementing the recommendations also served (NS, physician; CSAP, physiotherapist; AL, nurse-practitioner). A final team member served who has expertise in the various evaluative methods being applied (FA). We also solicited input from patient partners regarding whether the points to be conveyed at the bedside resonated with their information needs and preferences, was comprehensive, to ascertain if there were any omissions, and to ensure patient-centeredness. All 23 position statement-endorsing associations were informed about the plan to develop guideline implementation tool(s), with a request for eventual input and dissemination facilitation.

### Design and procedure

**Fig. 1:**
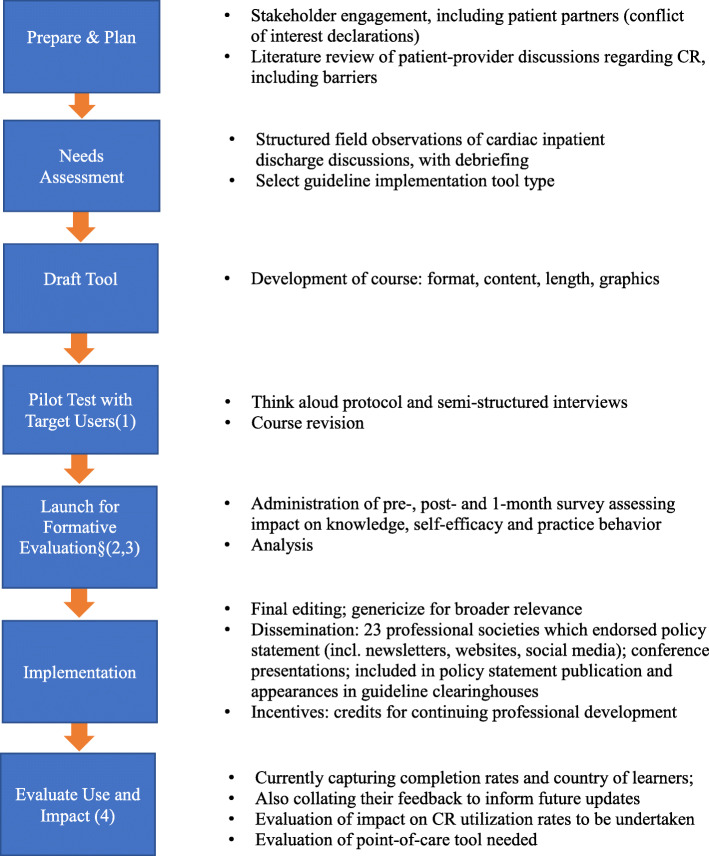
Process for development, evaluation and dissemination of the guideline implementation tool for promoting patient utilization of cardiac rehabilitation*. *steps based on cite [22–26]. §outcomes selected based on cite [27]. (Kirkpatrick’s levels for training evaluation) [27]

This was a multiple-method study, using a step-wise approach. Guideline implementation tool development process best practices were followed [22, 23], to consider needs for and barriers to implementation, determine the type of tool(s), develop it/them, evaluate and disseminate. The process is summarized in Fig. 1.

HCPs treating inpatients indicated for CR were recruited between September 2018 and May 2019 from two hospitals of an Academic Health Sciences Centre in Toronto, Canada (University Health Network; UHN), for the needs assessment (September–November 2018; structured observation), pilot test (November 2018–January 2019; interviews) and then evaluation (January–March 2019; prospective design). There are different CR referral processes on the various cardiac units at the hospitals; for some it was an electronic systematic referral, on another referral is included on the paper-based discharge order set, and on others there is no systematic process in place and hence physician referral is ad-hoc. Note that CR services are covered by government healthcare sources in Ontario.

#### Needs assessment

First, literature regarding patient-provider discussions about CR was reviewed, including barriers [28, 29]. Experts on patient-provider discussions regarding CR were consulted.

Second, cardiac HCPs were observed (structured) [30] during inpatient-provider discussions regarding discharge, to learn what information was being conveyed regarding CR (and not), and what questions inpatients often have about CR, to ensure providers have the answers in the future (Additional file 1: Appendix 1). To decrease the risk of reactivity, HCPs were informed we were interested in patient-provider communication regarding discharge instructions (CR was not mentioned until after the observation). The observer stood against a wall at some distance from the patient and HCP with a clipboard, and did not speak during the interaction.

After the observation, the observer debriefed with the HCP to get further detail regarding what information they felt they were lacking with regard to CR, and how they can be supported to discuss CR with patients. All observations and a discussion summary were recorded in writing immediately.

#### Implementation tool type and development

A review of guideline implementation tool types [31, 32] was considered by the team. Results of the literature review and structured observation were discussed with the team, and expert opinion was also considered to decide on the type of tool(s). Development ensued in accordance with best practices [22]. Input from patient partners was sought, and incorporated.

#### Implementation tool pilot test: think aloud protocol and semi-structured interview

Once developed and hosted on UHN’s eLearning centre, inpatient cardiac HCPs were recruited to view the online course (including the pre and post-course survey), in accordance with Level 1 of Kirkpatrick’s model (reaction) [27]. It was pilot-tested with the intended audience using a think aloud protocol (TAP) with subsequent semi-structured interviews (i.e., retrospective questioning for triangulation), until saturation was achieved. This was undertaken in person at UHN. The purpose was to determine whether the drafted online course was applicable to target HCPs / realistic, met their information needs, was an acceptable length, to get input on graphics / visuals, ways to promote implementation of the ideas at the bedside, and how it could be revised to better meet their needs. Results informed refinement before launching the course.

The instructions for the TAP are shown in Additional file 1: Appendix 2. The encounters were audio-recorded, with permission. They were transcribed verbatim, except to preserve anonymity. The senior investigator (SLG) attended the first few pilot tests for training purposes, and to finalize the drafted TAP protocol and semi-structured interview guide.

#### Implementation tool finalization and soft launch

Results of the pilot-test were used to finalize the course. It was launched for all users at UHN.

#### Formative evaluation: survey

HCPs were surveyed to test whether knowledge, attitudes, confidence/self-efficacy and practice / behaviour (e.g., if HCPs provided materials like pamphlet or handouts to patients about CR to take home) changed following completion of the course. These outcomes were chosen based on Kirkpatrick’s model of training evaluation (level 2, learning) [27]. The questionnaire was administered online using Google forms: (1) before viewing the course; (2) immediately after viewing the course; and (3) 1 month later, via email. HCPs were emailed on several occasions with reminders to complete the 1-month post-course survey if they had not done so, to optimize response rate.

### Participants: recruitment and sample size

For each element of the project, participants consisted of acute cardiac care providers (e.g., nurses / nurse-practitioners, physicians, physiotherapists) on wards treating patients indicated for CR at UHN (e.g., short stay unit for percutaneous coronary intervention, cardiovascular surgery unit, general cardiology ward). There were no exclusion criteria.

#### Structured observation

To recruit for this initial needs assesment, all HCPs in the cardiology program were contacted through email by the clinical director, with a request to be observed during patient interactions regarding discharge (CR was not mentioned). Unit nurse managers also identified some staff to approach. Attempt was made to observe HCPs on several cardiac wards. The plan was to observe interactions until no novel observations were made.

Observations with patients who were eligible for CR (see indications and exclusions in policy statement) [15, 16] and who were soon to be discharged were undertaken. On the day of observation, HCPs approached patients in their circle of care without the observer present, to ask for their voluntary consent that an observer be present during the discharge discussion. Patients were informed that the observer was recording information about the HCP provision of discharge information, and only any questions or issues the patient raised would be notated (the rest of the observation pertained to the HCPs), and that their identity would remain anonymous. Willing patients provided verbal informed consent.

#### Think aloud protocol and semi-structured interview

After the tool was developed, eligible HCPs were contacted through email by the senior investigator (SLG), with a request to preview the drafted online course and provide input. Recruitment was targeted to solicit feedback from several relevant disciplines, with emails sent to physicians, nurses and physiotherapists. Sample size was determined by saturation; they continued until no novel input was received.

#### Survey

After the course revision and launch, eligible HCPs were contacted through email with a request to complete the online course, with the surveys. The emails were sent by the clinical director, and the senior investigator (SLG) later followed-up. The new course was also advertised in the monthly cardiology and cardiovascular surgery email blast. We also attended team meetings on the cardiac wards to promote the course. The clinical director offered a pizza lunch to the cardiac ward with the highest completion rate.

### Measures

#### Needs assessment: structured observation

The observer used a checklist (Additional file 1: Appendix 1) to record observations and short descriptions of the interactions. The checklist was developed by CSP and SLG, and pilot-tested in 2 interactions. Some revisions were made. The senior author observed the first few observations and subsequent debriefings, to provide feedback for training purposes. The senior author independently completed the observation checklist, and discrepancies were discussed with CSP. This was repeated until no further discrepancies arose following an observation.

#### Pilot-test: think aloud protocol and semi-structured interview

The TAP and interview guide are shown in Additional file 1: Appendix 2. The TAP was performed using best practices [33]. The semi-structured interview guide was developed by CSP and SLG, and pilot-tested as outlined above.

#### Formative tool evaluation: survey

The surveys administered at each point are shown in Additional file 1: Appendix 3. They consisted of multiple choice and true-false questions, as well as items with a 5-point Likert type scale for responding. They assessed the basic characteristics of the HCP (e.g., profession), as well as their CR knowledge (e.g., how familiar HCPs were with what is offered and delivered to patients in CR) and attitudes, self-efficacy in discussing CR with inpatients(e.g., how confident HCPs were addressing barriers patients raised regarding CR attendance), as well as their practices (e.g., giving CR program pamphlets to patients; Table 2).

### Analyses

#### Observation

SPSS version 24.0 was used for quantitative analysis. Elements of the observation coded as present or absent were described using descriptive statistics. Analysis of the qualitative data involved bringing order and structure to the information recorded to inform development of the online course [34].

#### Think-aloud and interviews

The transcripts of the TAP and subsequent questioning [35] were segmented into sensible chunks or communication units, which were coded, all by the first author [36, 37]. The coding of the TAP speech focused on thoughts reflecting ways in which the course could be improved (researcher inference; literal as much as possible), and of the interviews focused on validating interpretation of the think-aloud utterances, as well as extracting additional suggestions relating to how the course could be improved [38]. Initial coding by CSP was reviewed and discussed with the senior author, who was there for the initial interviews and reviewed the transcripts. Final coding / thematic content analysis was discussed between researchers to determine the course of action for revising the online course.

#### Survey

SPSS version 24.0 was used for analysis. All surveys were included. Descriptive statistics were used to describe the sample, as well as survey responses. Pre- and post-course survey responses were compared using paired t-tests or chi-square analyses as applicable (repeated measures analysis of variance was planned, but the sample size for the survey 1-month post-course was insufficient).

## Results

### Needs assessment: structured field observation

Seven HCPs (all nurses) were observed (8 interactions); 5 (71.4%) were female. In most interactions, HCPs were rushing, to complete their “tasks”. Family or informal caregivers were present for 5 (62.5%) interactions. Five (71.4%) HCPs knew whether their patient had been referred or was going to be referred.

Five (62.5%) HCPs initiated a dialogue about CR with a patient; however, the dialogue lasted an average of 12 s and lacked detailed information. No patients raised CR. In all interactions, CR was raised after the discharge instructions, at the end of the interaction.

In 1 (20.0%) of these 5 interactions, HCPs explained what CR is (e.g., consists of education and physical exercise), and none of these interactions was the information conveyed all accurate. In no interactions did HCPs explain why the patient was being referred, in 1 (20.0%) interaction the HCP mentioned some of the benefits of CR (i.e., “faster recovery”, “get back on their feet”). In 2 (40.0%) interactions, the HCPs provided strong and explicit positive endorsement of CR, which the observer rated as a mean of 4.5 / 5 (Additional file 1: Appendix 1, item 8). In 1 (20.0%) interactions the HCP explained next steps to enroll (see below).

In only 1 (20.0%) interaction where CR was raised did the HCP invite questions about CR; and in 1 (20.0%) patients raised questions. Some patients asked about when the program would start and whether family members could attend, yet most HCPs did not know the answers. In 2 (40.0%) interactions barriers were raised, and 2 HCPs discussed ways to overcome them (e.g., HCP explained patient would be directed to CR program closest to home); in most cases, barriers were not sufficiently addressed. Overall, there was 2-way discussion about CR in 1 of the 8 encounters, and patients were provided a means to find out more information in 5, however this consisted of a brochure included among other brochures provided to patients at discharge, and the HCPs did not refer patients to it specifically.

Most commonly the HCPs gave the following 2 points when discussing CR: “The cardiac rehab program will call you in 2 weeks, and you will be referred to the program closest to home.” One HCP stated that the patient did not have to attend CR if they did not want to.

In an observation where CR was not initially raised, a senior nurse was training a new hire on how to go through the discharge summary with patients; the nurse lacked information about who should refer the patient to CR and stated: “If a patient asks about CR, just tell them that the family doctor will decide if they need to go, and they will be referred [to a site] close to home.” During the observation the nurse trainee did not mention CR to the patient, and after debriefing with the researcher, the trainee felt compelled to go back and explain about CR to the patient, who asked many questions and seemed interested and likely to attend. In the other observation where it was not raised, the patient had an interpreter because he could not understand the English language; during the observation the HCP failed to mention CR to the patient. When asked the reason during debriefing, the HCP responded: “I just forgot to mention CR.”

During the debriefing after the observations, most staff seemed aware of the importance of discussing CR participation with their patients. Overall, the observations revealed that HCPs are insufficiently discussing CR with their patients, wanted to know about who was eligible, and what were valid reasons patients should not go as well as what was not.

### Implementation tool development

Based on the results from the structured observation, for policy statement recommendation implementation support, the team elected to develop training material for HCPs [31]. It was decided to develop an online course given how busy inpatient HCPs are, and that they complete online courses annually as a requirement for continuing professional education. The course was sponsored by the hospital’s CR program, and built by an eLearning and instructional design specialist from UHN in alignment with their best practices (Fig. 1).

The training course was designed to inform inpatient cardiac care providers about: (1) what is cardiac rehab (and provide a corresponding patient handout); (2) the benefits of participation; (3) the importance of, and how to provide a positive endorsement regarding participation to patients; and (4) the importance of letting patients ask questions and discuss any barriers they may have. Input was also gathered from patient partners and other stakeholders (e.g., policy statement-endorsing societies) [15, 16] on the main points to convey. With the patient partners, we considered how to convey risk associated with non-participation when stating CR benefits, and also considered evidence on how best to do this to encourage patient enrolment (e.g., gain frame – 25% less likely to die if go to CR) [39].

### Pilot test: think Aloud Protocol & Semi-structured Interviews

The TAP and interviews were conducted with female HCPs (2 nurse managers, 2 cardiology fellows [MD], and 1 nurse-practitioner), and averaged 22 min. Data collected from the TAP and interviews suggest that HCPs were satisfied with the content and length of the course. Themes are shown in Table 1 with examples and corresponding revisions made to the online course.

**Table 1:** Selected coding from think-aloud protocol and subsequent interviews, with corresponding changes made to course

	Supporting segments / units (HCP #; *italicized text is excerpt from course on which HCP is reflecting*)	Changes made to online course
1: Details about CR delivery	*Over approximately 5 months, patients participate in sessions approximately 2 times per week covering guideline-based “core components”, including: education, exercise and counselling.* “I think when providers and patients think of CR they often think they’re just going to exercise. So, I liked that this breaks down that you’re also going to get counseling and you’re also going to get education on your condition. So that’s good (HCP 3).” *Factors that should not impact referral to CR. Ambulation: These patients can still benefit from other components of CR, such as patient education, dietary counseling and stress management.* “So I think that’s big because I know that I have a bias in my mind if a patient isn’t able to exercise or isn’t able to ambulate well. I often think of the benefit of rehab being minimal, but again, that’s the bias of me thinking about it more as exercise as opposed to the other components. So, I like that (HCP 3).” *Recognizing patients who are indicated for cardiac rehab or who meet indications for Cardiac Rehab and understand how they are referred at UHN.* “Referral is a big part because even though we know CR exists, sometimes the referral process is a little bit like, well, how do we get patients there?... It’s important we understand how that is done (HCP 3).” *First language spoken- interpretation services are available.* “I did not know that. I like that it shows it’s really an all-inclusive process and that, as a rehab center we really try and accommodate people’s different levels of abilities. So, I liked that (HCP 3).” *Patients will be triaged closest to home.* “So, the actual CR here, will figure out where they live and figure out where is the best rehab center for them? Okay, great (HCP 3).”	–
2: Good and not good candidates for CR	*Poor Candidates for CR – serious mental illness.* “Serious mental illness… I don’t really classify depression as serious (HCP1).” *Poor Candidates for CR section.* “I think most of these (not good candidates) should be obvious, but I think it’s helpful to reiterate to us, as clinical practitioners, you don’t want to send someone to rehab that it’s going to be a dangerous process for them (HCP 3).”	**Added pop-up detail:** Serious mental illness, not including depression or anxiety
3: CR model	*What is CR? CR is an outpatient chronic disease management program, addressing all guideline recommendations for secondary prevention. Recommendations that you provide inpatients are reinforced.* “I think in this section about what CR is, the thing that comes up clinically a lot is how flexible is it? What times of day is it? Like if they’re working, is it still an option or that sort of thing…These are the questions that I don’t always have the answers to (HCP 2).” *Barrier #3: Patient is returning to work, lives out of town, or has no way to get to CR. Explain to the patient: Most CR programs offer “home-based” models.* “Home-based models? Is this where like they would give an exercise prescription so that they could come in less often? I think like that would play well into what we were talking about before too, with like how flexible it is versus, like do you have to come in two times a week and what hours of the day it is and that sort of thing... Because that does come up a lot, especially for younger patients (HCP 2).” “Oh, okay… Like over the internet or something like videos? I actually didn’t know that education support can be provided over the phone. Very nice! (HCP 5).” “I didn’t know that there was a home-based model from the get-go. I always thought they had to do the five months in rehab. Like in the physical place and then they could have their exercise prescription and be supported with their home-based? So, that’s good to know (HCP 3).”	**Added text:** Patients participate in sessions, in-person or on the phone, covering guideline-based “core components”, including education, exercise and counseling. The number of sessions or calls varies by program (on average twice per week over 5 months) and are offered when convenient to the patient. Family members are welcome to attend as well.
Theme 4: Patient safety concerns	*How patients are referred at UHN.* “The biggest issue with my team is that the interventionalists are not highly convinced that patients should exercise and what length of time after their event they should start to exercise. Is every rehab supervised differently? Do they all have physicians? Who is responsible for the patient? (HCP 1).”	**Added phrase:** The patient will not undergo any exercise stress testing until they have been pre-screened and only under the supervision of a physician. Safety should not be a concern.
Theme 5: CR discussion – provider type	*Nurses are primarily responsible for initiating this discussion.* “I think that’s great that nurses are responsible, but I also think that we as residents and physicians when discharging the patient should put a positive vote in for the program as well. We have evidence that shows patients are more likely to get engaged in things that physicians recommend. So, I think, we need to do a better job of promoting it as well (HCP 3).” “Is it that we’re saying that nurses should be the first point of contact in this discussion? Is it that they should start this discussion before a physician thinks that it is appropriate? (HCP 4).” “I think primarily nurses might not feel comfortable having that initial discussion with someone, let’s say with heart failure, who doesn’t fit the criteria or someone who’s being admitted with some rhythm abnormalities. When is it safe to have that discussion? So, I almost feel like the first person should be the most responsible physician or clinician should have that initial conversation and nurses certainly can help (HCP 4).”	*Nurses sentence was removed, and sentence was added:* Often, providers are not sure who is going to discuss CR with patients, so no one does. Ideally, the physician should inform the team and patient that the referral is being made and nurses and allied HCP should reinforce this message by informing patients more fully about getting started.
Theme 6: CR participation barriers	*Key points for discussion: discuss how to overcome any raised barriers to entering the program* “I was always kind of under the impression that, you know, if I say to a patient that the CR will call them. If there are any barriers they can be addressed with the person over the phone. I don’t know exactly what their capabilities are… I usually just encourage them to work at it with the CR. Maybe that’s wrong what I’ve been doing. Because I don’t think I’m very well equipped to overcome some of the barriers (HCP 2).” “A big barrier for patients is the language and cultural. You know in some cultures, women traveling long distances alone and things like that…or I don’t think my mother would benefit from that because she doesn’t really speak English that well or we can’t get her there and that type of thing (HCP 4).” “I think the barriers are good barriers that were identified that our patients would have (HCP 5).”	
Theme 7: Request for additional information	*Benefits of CR include: Reducing cardiovascular death and re-hospitalization by 20%* “It’d be nice to see some of this data. Reducing death and rehospitalization... It would be good to see some of the data (HCP 2).” “So, I think these last two points (Reducing cardiovascular death and re-hospitalization by 20% and significantly improving the patient’s quality of life), um, I think a lot of healthcare providers will be impressed by the statistics. Does that mean it needs to go at the top?... I think maybe putting the point about and cardiovascular death and re-hospitalization higher up or maybe bolded. I think that could reinforce (HCP 3).”	Figure with forest plot and manuscript citation was added, and the phrase “Reducing cardiovascular death and re-hospitalization by 20%” was bolded.
Theme 8: eLearning module feedback	*Tools to support your discussion. Click the resources to the right for tools to support your discussion with the patient regarding CR.* “Oh, so this is like a pamphlet you can give to the patient to help them understand in writing what it is you’ve talked about? I like this because often I find when we give patients information at the bedside, they retain maybe 10 or 20% of it. I liked that they have option for something to take with them (HCP 3).” *Summary: You have reached the end of the Promoting Patient Participation in CR eLearning course.* “The course length is fine, it’s good, it’s not too much (HCP 1).” “The course was very good (HCP 1).” “Can I add that I really liked the length. Like I think that it’s important that it’s not too, too long. With the addition of a couple of slides maximum, like a little bit extra information that I think is high yield, I wouldn’t change it very much (HCP 2).” “I thought it was concise. It wasn’t too verbose. I thought it was really well done (HCP 3).” “I think it was decent. It was good. I think appropriate. Ten to 15 min is fair. (HCP 4).” “The course length wasn’t bad at all. It was pretty fast (HCP 5).”	
Theme 9: Course improvement	*What suggestions do you have for us to improve the course? How can we better support providers such as yourself to promote CR to your patients?* “I think the biggest thing is giving them (providers) tools. They need a lot of education about the importance of CR (HCP 5).” “I think more and more today people are doing or using those tools electronically. So, you know, like the little pocketbooks, the little, ACLS resuscitation cards that we use, and I just have those on my phone and I saved them as different files on my phone. But even if you had a pocket card, I think certainly the older generations would like that. And then if you don’t want it in physical form and you only want in digital form, you could always just take a picture and have it as a file on your phone. So, I like that. (HCP 3).” “I think most people are very used to just having brochures in front of them and using that as a method for ensuring that they’re getting everything that they’re actually capturing everything. Something visual is important when we’re talking about, especially if it’s nurses, clinicians, that you just want to have that in front of you... To make sure you don’t miss anything important (HCP 4).” *Do you think you will remember the points to discuss with patients? (referring to the Points to Discuss slide)* “Well, I guess it’s not standing out to me from the presentation which are key points for discussion, I know what I’ll usually tell patients, but I’m not sure… There’s something about it that’s not very memorable. Maybe create a handout… You know, what I think works well is things like little cards that people can attach to their badge… easy to carry around and keep on you or like in a lab coat. You know what I mean, like really small and portable. Or do a handout, but you know what, but a handout is always tricky. Like we’ll just throw them out. You know, what I think works well is things like little cards that people can attach to their badge. I’ve gotten like stroke handouts and things like that are actually easy to carry around and keep on you. Or like in a lab coat. But if it’s this size and it says like, you know, it’s like 12 words, but it says like, what is CR, benefits? You know what I mean, like really small and portable. Then I think that it works better, but truly this slide was not memorable to me (HCP 2).” “What you could do instead of creating a handout it could be a pocket card that you give out to people? When you complete the course, you can enter your email address and then you email the recipient a PDF of that pocket card? (HCP 3).”	A PDF tool with key points for discussion was created, which learners could download, and keep it in their phones.

**Fig. 2:**
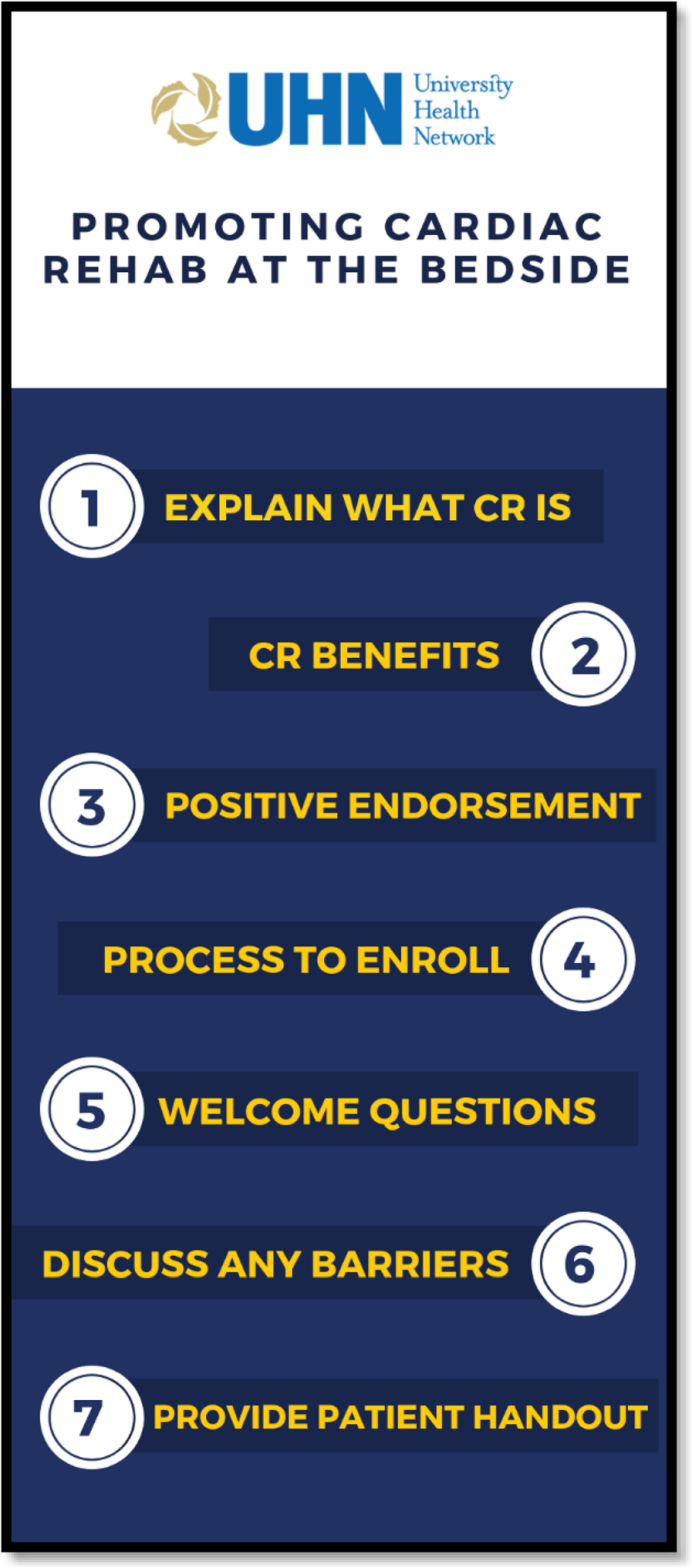
Point-of-care tool: Key points for patient-provider discussion. CR = cardiac rehabilitation

*CR* cardiac rehabilitation, *HCP* healthcare providers;

Revisions included providing evidence (i.e., forest plot and citation) [7] on the benefits of CR, as well as clarifying that pre-CR stress tests are performed under physician supervision and only after patient evaluation for readiness/safety. Additionally, HCPs did not remember the key points to convey to patients, so we developed and embedded a “key points” handout learners can print to use at the point-of-care (Fig. 2).

### Formative tool evaluation: knowledge, attitudes, self-efficacy, practice

Twenty-four HCPs (20 registered nurses [83.3%], 1 nurse-practitioner [4.2%], 1 physiotherapy assistant [4.2%], and 2 other HCPs [8.3%]; 23 female [95.8%]; mean age = 36.4 ± 11.6 years) viewed the online course and completed the surveys (retention shown in Table 2).

**Table 2:** Survey responses by assessment point

*n* (%) or mean ± Standard Deviation	Assessment Point		
	Pre-Course 24 (100.0%)	Post-Course 21 (87.5%)	1 Month Post-Course 9 (37.5%)
**Knowledge**			
How familiar are you with what is offered and delivered to patients in CR?†	2.71 ± 0.95	4.10 ± 0.62***	3.78 ± 0.67
Do you know how to ensure eligible/indicated cardiac patients in your care are referred to CR? (% yes)	9 (37.5%)	21 (100.0%)§	9 (100.0%)
Do you perceive you have all the information you need to comprehensively discuss CR at the bedside with your patients?¥	2.25 ± 0.90	3.90 ± 0.54***	2.78 ± 0.97
Which of the following patients are not good candidates for CR?			
STEMI patient who is depressed	4 (16.7%)	3 (12.5%)	4 (44.4%)
Ventricular arrhythmia patient who is depressed°	12 (50.0%)	17 (81.0%)	8 (88.9%)
NSTEMI patient who lives outside of the city	5 (20.8%)	0 (0%)	0 (0.0%)
Patient with decompensated heart failure that lives outside of the city°	12 (50.0%)	15 (71.4%)	3 (33.3%)
Older NSTEMI patient without a spouse / informal caregiver to help with CR transportation	7 (29.2%)	1 (4.8%)	0 (0.0%)
**Self-Efficacy**			
How confident are you that you can address any barriers patients raise regarding CR attendance? □	2.42 ± 0.88	3.76 ± 0.54***	3.11 ± 0.60
How confident are you in answering questions patients raise about attending CR?□	2.29 ± 0.95	3.67 ± 0.58***	3.44 ± 0.88
**Attitudes**			
How important is it to you to provide information about CR to patients before they are discharged? ∞	4.13 ± 0.95	4.62 ± 0.59*	4.22 ± 0.67
**Practice**			
Do/will you provide any materials to patients about CR to take home with them (e.g., pamphlet or handout with weblink)?		§	
Yes, most of the time	3 (12.5%)	21 (100.0%)	5 (55.6%)
Sometimes	5 (20.8%)	–	1 (11.1%)
No	16 (66.7%)	–	3 (33.3%)

*denotes significant difference between pre and post-course scores tested via paired t-test or chi-square, as applicable: *p < .05, ***p < .001. Differences from the 1-month post-course scores were not tested due to the small sample size

§differences from pre to post-course could not be tested as some cells had zero counts

†scores range from 1 “I am not familiar with CR” to 5 “very familiar”

¥scores range from 1 “No” to 5 “Yes, I definitely have all the information I need to discuss CR”

□scores range from 1 “Not at all confident” to 5 “very confident”

∞ scores range from 1 “Not at all important” to 5 “very important”

°these patients would not be good candidates

□intentions only at this point

CR: cardiac rehabilitation; STEMI: ST-elevation myocardial infarction; N-STEMI: Non ST-elevation myocardial infarction

When asked pre-course whether their patients were generally referred to CR, 7 (29.2%) HCPs reported that patients are referred most of the time, 14 (58.3%) reported sometimes, and 3 (12.5%) indicated they are not referred. When asked whether they discuss CR with patients, 9 (37.5%) HCPs reported most of the time, 7 (29.2%) sometimes, and 8 (33.3%) never.

Survey responses are displayed in Table 2 by assessment point. As shown, viewing the online course resulted in significant increases in knowledge of what CR entails, having sufficient information to comprehensively discuss CR with patients, self-efficacy in addressing patient questions about CR and barriers, and attitudes toward discussing CR with patients. In terms of knowledge regarding types of patients that are eligible, pre-course HCPs were accurate for a mean of 3.33 ± 0.87 of the 5 patient profiles, post-course HCPs were accurate for a mean of 4.33 ± 0.86 of the 5 profiles (paired t = 3.90 *p* = .001), and 1 month later for 3.78 ± 0.66. Differences in practice could not be tested. Overall, for all items that could be tested, significant improvements were observed following viewing the course.

## Discussion

Guidelines and Position Statements can play an important role in health policy formation and health care delivery [40]. However, the development of guidelines with recommendations is insufficient to change practice; the recommendations must be implemented. A multitude of determinants influence if recommendations are implemented, at the guideline, clinician, patient, organization and healthcare system levels [23]. To our knowledge, there are no other implementation tools that are evidence-based which address how to increase CR utilization.

After an extensive literature review and needs assessment, a novel guideline implementation tool was developed to promote patient-provider bedside discussions regarding CR. The online course was pilot-tested with acute cardiac care providers. Subsequent evaluation revealed that viewing the course resulted increased CR knowledge, self-efficacy regarding discussing CR with patients, and more positive CR attitudes among HCPs. A point-of-care tool was also developed to support HCPs in having a fulsome discussion with patients at the bedside.

With these positive results, we went on to the implementation phase of the process (Fig. 1). The online course has been genericized for a broader audience of inpatient cardiac care providers globally. This involved primarily removing institution-specific referral and CR program information. The course is available here: http://learnonthego.ca/Courses/promoting_patient_participation_in_CR_2020/promoting_patient_participation_in_CR_2020EN/story_html5.html (there are also French and Portuguese translations available; Chinese and Spanish translations are currently underway). We have applied for and secured continuing education credits for course completion (http://ccs.ca/en/professional-development/programs-and-events).

Thus, we are now seeking to inform our target audience of the availability of the online course, to promote wide learning. We are submitting the policy statement to guideline clearinghouses (e.g., https://guidelines.ecri.org/brief/1547#implementationTools;https://joulecma.ca/cpg/search/view/19420; https://g-i-n.net/library/relevant-literature/promoting-patient-utilization-of-outpatient), and including this as an implementation tool. We have asked the 23 position statement-endorsing societies and 39 ICCPR-member societies to disseminate the course to their members; they are doing this via email, websites and social media.

### Directions for future research

As per the final step of the process in Fig. 1, the genericized course require evaluation in a broader, larger sample. The evaluation should include investigation of change in practice (i.e., occurrence of discussions; i.e., Level 3 of Kirkpatrick’s model- behavior) [27], the quality of CR discussions (e.g., structured observation pre and post-course viewing), and impact on CR utilization (i.e., Level 4 of Kirkpatrick’s model - results) [27]. Many CR associations have utilization quality indicators which could be used to quantify impact [41]. Impact on HCP practices and quality of CR discussions over the longer-term post-course also should be assessed; there was insufficient data in the current study to even determine effect 1-month post-course, but what data are available suggest there is some decay over time without reinforcement.

As a requirement for being an accredited learning activity for continuing education credits, the genericized course does have a pre and post-course knowledge survey as well as evaluation / feedback on the course, which will be collated in future. We are also capturing country of origin of learners, and monitoring usage / uptake.

Other important avenues for future research include investigating inpatient CR information needs and preferences, such that a more standardized discussion could be specified for HCPs. This should then be evaluated, in terms of acceptability by patients, satisfaction, and ultimate CR utilization. The point-of-care tool could be revised based on patient input, and with evidence of impact on CR use. There truly is little evidence or guidance regarding the content of CR discussions, and based on our observations it seems some of the discussions that do occur may dissuade patients from attending or reduce their likelihood of enrolling. A question prompt tool for patients may also be helpful [42].

In the United States, the Agency for Healthcare Research and Quality (AHRQ) recently awarded a contract for implementation of systematic referral with a “liaison” discussion at the bedside in 100 hospitals (https://www.ahrq.gov/pcor/dissemination-of-pcor/cardiac-rehabilitation.html). Participating hospitals will be supported in a learning community, and will work on an aspect of implementation each month over the course of a year. This reinforcement may ensure sustained implementation (versus the decay we seem to have observed by 1-month post-course viewing). The online course may be useful in educating HCPs regarding bedside CR discussions. To be successful, it is helpful that the project ensures the referral itself, but also patients should be consulted about their needs and preferences for CR information at the bedside (see above), and inpatient units need to collaborate closely with the CR programs to which they refer to ensure they can accept additional patients (or increase their capacity if not). While single-faceted and passive approaches can be effective [31], a systems approach, with tools for all aspects of the process (toolkit), is recommended to achieve CR enrolment targets [15, 16].

Caution is warranted in interpreting these results. The study was single centre, and therefore it must be tested whether findings generalize, particularly to non-academic centers. At least the course was piloted on various types of cardiac wards. Second, unfortunately only nurses were willing to be observed for the needs assessment, but the team / planning committee did represent the types of HCPs targeted for the course. Third, during the structured observation, some HCPs might have altered their behavior due to an awareness that they were observed; however, they were not informed which aspects of their patient interaction was being evaluated. Given the low quality and quantity of the CR-specific content observed, this is likely not a significant concern. Finally, the sample size was small for the survey, and retention low.

## Conclusion

The online course developed is the first available to our knowledge to educate HCPs regarding communication at the bedside to encourage patient utilization of CR, as per policy statement recommendations. The results of the evaluation suggest that HCPs who completed the online course had increased CR knowledge, self-efficacy and more positive attitudes. These preliminary results suggest broader dissemination and evaluation is warranted. It is hoped this tool can support inpatient cardiac care units to achieve 70% CR enrolment of their patients, so the high burden of CVD can be ameliorated.

## Supplementary information

**Additional file 1: Appendix 1.** Coding Guide for Structured Observation of Patient-Provider Interaction. **Appendix 2.** Think Aloud Protocol and Semi-Structured Interview Guide for Tool / Course Pilot Test. **Appendix 3.** Online Course Surveys.

## Data Availability

The datasets used and/or analyzed during the current study are available from the corresponding author on reasonable request.

## Abbreviations

AHRQ: Agency for Healthcare Research and Quality
CR: Cardiac rehabilitation
CVD: Cardiovascular diseases
HCP: Healthcare provider
HCPs: Healthcare providers
ICCPR: International Council of Cardiovascular Prevention and Rehabilitation
TAP: Think aloud protocol
UHN: University Health Network
