# Supplementary material for: Implementing recommendations for inpatient healthcare provider encouragement of cardiac rehabilitation participation: development and evaluation of an online course
Source: BMC Health Serv Res. 2020 Aug 20;20:768. doi: 10.1186/s12913-020-05619-2 (PMC7439558; doi:10.1186/s12913-020-05619-2)
Supplement: Supplementary file 1 — Additional file 1: Appendix 1. Coding Guide for Structured Observation of Patient-Provider Interaction. Appendix 2. Think Aloud Protocol and Semi-Structured Interview Guide for Tool / Course Pilot Test. Appendix 3. Online Course Surveys. [file 12913_2020_5619_MOESM1_ESM.docx]

**Appendix 1**. Coding Guide for Structured Observation of Patient-Provider Interaction

Healthcare provider #: ___________

Healthcare provider discipline: _____________________________________

Site and cardiac ward: ____________________________________________

Patients day of hospital stay: _______ of ______ (if known)

Date and time: __________________

|  | Element | Present/  Absent | Comments/observations  (including any inaccurate information conveyed) |
| --- | --- | --- | --- |
| 1 | Initiated dialogue about Cardiac Rehabilitation (CR) referral with patient or family member |  |  |
| 2 | Were any family members or informal caregivers present? |  | *If yes, type (if known) & #* |
| 3 | CR discussion embedded with other conversation? (for context) |  | *Make notes about what else was discussed with patient during encounter* |
| 4 | Explained what CR is |  | *Was it accurate?*  *Summarize description here* |
| 5 | Explained why patient is being referred -i.e,. all patients with their heart condition are to be referred |  |  |
| 6 | Mentioned some benefits of CR participation |  | *Specify which ones* |
| 7 | Provided strong and explicit positive endorsement of CR participation to patient or family member |  |  |
| 8 | Rate from 1 (negative) to 5 (very positive) how positive the endorsement seemed to you: |  |  |
| 9 | Explained that the cardiac rehab program will call the patient at home a few days after their discharge -i.e., what are the steps to enroll |  |  |
| 10 | Invite patient / family member questions about CR | - Yes - No - Not applicable because the patient/family raised questions spontaneously | *If yes, state what was asked*  *Were they answered satisfactorily?*  *Summarize responses* |
| 11 | Discussed how to overcome any raised barriers to entering a program | - Yes - No - Not applicable because no barriers raised | *Summarize.*  *Were barriers addressed / mitigated?*  *How?* |
| 12 | Was there 2-way discussion about CR |  |  |
| 13 | Discussed or provided materials / tools about CR program (e.g., brochure, website) |  | *Specify what provided* |
| 14 | Did the healthcare provider know if the patient was referred or not? | - Yes, HCP knew pt was referred - Yes, HCP knew pt wasn’t referred or pt was not a good candidate - No, HCP didn’t know if pt referred | *Did it seem the HCP knew how pts were referred and who did it?*  *How? Conveyed to patient? Checked where / with who?* |

Length of CR-specific discussion:________ minutes: _______ seconds.

HCP=healthcare provider; pt=patient

NOTES:

1. Reflections on whether it seems patient is likely to attend: ____________________________________________________________________________________________________________________________________

**Appendix 2**. Think Aloud Protocol and Semi-Structured Interview Guide for Tool / Course Pilot Test

“Hello, my name is Carolina Santiago; I am a Physiotherapist and a PhD student at York University. My supervisor is Sherry Grace, a Scientist in Cardiac Rehabilitation at University Health Network (UHN).

We have developed an online course to support inpatient cardiac healthcare providers such as yourself to promote cardiac rehab use in your patients at the bedside. Your input will help us improve and finalize the course before we launch it. We want to make sure it is as useful for inpatient care providers as possible.

First of all, thank you for agreeing to help us. We will do 2 things: (1) you will watch the course and state your impressions as you go along, and (2) answer a few questions at the end. This should take around 20 minutes.

The course has been approved by UHN, and will be hosted though our learning management system MyLearning. If effective, we will circulate it more broadly.

As you watch the course, think out loud. By that, I mean while you are going through the slides, I want you to state what you’re thinking as you go along. For instance, if the content is unclear or needed information is missing, please say those things out loud. Please be forthright so we get the most input we can to improve it.

We would like to test the course under real-world circumstance, so we will pretend that you are on your own. I will be making notes as you go along.

Is it okay if I record our discussion? I will be sure not to link your identity to the recording. We have an ethics waiver to evaluate this.

Do you have any questions before we begin?

RECORD.

Post-Viewing Questions for Semi-Structured Interview:

Thank you. Your insights have been very helpful. What questions do you have about the course or its’ contents before I ask mine?

1. Was the content applicable to your reality on the cardiac ward? Were the recommendations for promoting patient use of cardiac rehab realistic?
2. Do you think you will be able to use and apply the information from the course to talk to your patients about CR? In what ways?
   - If not, why not? Is there different information you would need?
3. Did the 3 sections (i.e., what is CR, how referral is made, and what to say to patients) make sense and flow?
4. How was the length of the sections and duration of the course?
   - Is there any information you think that was not necessary to include?
   - Any information that was missing that would help you talk to your patients about cardiac rehab?
5. Any of the graphics not resonate with you? Things we should revise?
6. Was there a part of the course that made you feel more inclined to promote CR to your patients?
   - Was there anything in the course that dissuaded you from wanting to talk to your patients about CR?
7. Do you think you will remember the points to discuss with patients? How can we promote implementation of the recommendations with patients at the bedside?
8. What suggestions do you have for us to improve the course? How can we better support providers such as yourself to promote CR to your patients?
9. Lastly, is there anything else we should consider?

*[After completion of post-course questions]* OK, we’re finished. Thank you so much for your time. Your input was invaluable.

**Appendix 3**. Online Course Surveys

**Time 1: Pre-course**

Please complete the following quiz. You will be asked complete an adapted version of this quiz at the end of the course, and again in one month.

1. What is your profession?
   - Physician
   - Nurse-practitioner
   - Nurse
   - Physiotherapist
   - Other allied healthcare provider
   - Other (please specify: ____________________)
2. How familiar are you with what is offered and delivered to patients in cardiac rehabilitation (CR)?
   - Very familiar
   - Quite familiar
   - Somewhat familiar
   - Scantly familiar
   - I am not familiar with CR
3. Are eligible/indicated cardiac patients in your care referred to CR?

- Yes, most of the time
- Sometimes
- No

1. Do you know how to ensure eligible/indicated cardiac patients in your care are referred to CR?

- Yes
- No

1. Do you discuss CR participation with eligible/indicated patients at the bedside?

- Yes, most of the time
- Sometimes
- No

1. Do you perceive you have all the information you need to comprehensively discuss CR at the bedside with your patients?
   - Yes, I definitely have all the information I need to discuss CR
   - Yes, I have the information I need
   - I have most of the information I need
   - I don’t really have the information I need
   - No
2. Do you provide any materials to patients about CR to take home with them (e.g., pamphlet or handout with weblink)?
   - Yes, most of the time
   - Sometimes
   - No
3. How important is it to you to provide information about CR to patients before they are discharged?
   - Not at all important
   - Slightly important
   - Somewhat important
   - Quite important
   - Very important
4. How confident are you that you can address any **barriers** patients raise regarding CR attendance?
   - Not at all confident
   - Not very confident
   - Somewhat confident
   - Quite confident
   - Very confident
5. How confident are you in answering **questions** patients raise about attending CR?
   - Not at all confident
   - Not very confident
   - Somewhat confident
   - Quite confident
   - Very confident
6. Which of the following patients are **not** good candidates for CR? (check all that apply)
   - STEMI patient who is depressed
   - Ventricular arrhythmia patient who is depressed
   - NSTEMI patient who lives outside of the city
   - Patient with decompensated heart failure that lives outside of the city
   - Older NSTEMI patient without a spouse / informal caregiver to help with CR transportation

**Time 2: Post-course**

1. How familiar are you with what is offered and delivered to patients in cardiac rehabilitation (CR)?
   - Very familiar
   - Quite familiar
   - Somewhat familiar
   - Scantly familiar
   - I am not familiar with CR
2. Do you know how to ensure eligible/indicated cardiac patients in your care are referred to CR?

- Yes
- No

1. Will you provide materials to patients about CR to take home with them (e.g., pamphlet available through the weblink shown in the course)?
   - Yes, wherever possible
   - Sometimes
   - No
2. Do you perceive you have all the information you need to comprehensively discuss CR at the bedside with your patients?
   - Yes, I definitely have all the information I need to discuss CR
   - Yes, I have the information I need
   - I have most of the information I need
   - I don’t really have the information I need
   - No
3. How important is it to you to provide information about CR to patients before they are discharged?
   - Not at all important
   - Slightly important
   - Somewhat important
   - Quite important
   - Very important
4. How confident are you that you can address any **barriers** patients raise regarding CR attendance?
   - Not at all confident
   - Not very confident
   - Somewhat confident
   - Quite confident
   - Very confident
5. How confident are you in answering **questions** patients raise about attending CR?
   - Not at all confident
   - Not very confident
   - Somewhat confident
   - Quite confident
   - Very confident
6. Which of the following patients are **not** good candidates for CR? (check all that apply)
   - STEMI patient who is depressed
   - Ventricular arrhythmia patient who is depressed
   - NSTEMI patient who lives outside of the city
   - Patient with decompensated heart failure that lives outside of the city
   - Older NSTEMI patient without a spouse / informal caregiver to help with CR transportation

**Time 3: One month Post-course**

1. How familiar are you with what is offered and delivered to patients in cardiac rehabilitation (CR)?
   - Very familiar
   - Quite familiar
   - Somewhat familiar
   - Scantly familiar
   - I am not familiar with CR
2. Are eligible/indicated cardiac patients in your care referred to CR?

- Yes, most of the time
- Sometimes
- No

1. Do you know how to ensure eligible/indicated cardiac patients in your care are referred to CR?

- Yes
- No

1. Do you provide materials to patients about CR to take home with them (e.g., pamphlet)?
   - Yes, wherever possible
   - Sometimes
   - No
2. Do you perceive you have all the information you need to comprehensively discuss CR at the bedside with your patients?
   - Yes, I definitely have all the information I need to discuss CR
   - Yes, I have the information I need
   - I have most of the information I need
   - I don’t really have the information I need
   - No
3. How important is it to you to provide information about CR to patients before they are discharged?
   - Not at all important
   - Slightly important
   - Somewhat important
   - Quite important
   - Very important
4. How confident are you in addressing any **barriers** patients raise regarding CR attendance?
   - Not at all confident
   - Not very confidentbar
   - Somewhat confident
   - Quite confident
   - Very confident
5. How confident are you in answering **questions** patients raise about attending CR?
   - Not at all confident
   - Not very confident
   - Somewhat confident
   - Quite confident
   - Very confident
6. Which of the following patients are **not** good candidates for CR? (check all that apply)
   - STEMI patient who is depressed
   - Ventricular arrhythmia patient who is depressed
   - NSTEMI patient who lives outside of the city
   - Patient with decompensated heart failure that lives outside of the city
   - Older NSTEMI patient without a spouse / informal caregiver to help with CR transportation
